# Supplementary material for: Metal artifact reduction combined with deep learning image reconstruction algorithm for CT image quality optimization: a phantom study
Source: PeerJ. 2025 Jun 4;13:e19516. doi: 10.7717/peerj.19516 (PMC12145087; doi:10.7717/peerj.19516)
Supplement: Supplemental Information 5 [file peerj-13-19516-s005.docx]

**Supplemental Table 1** Statistical analysis of SNR under different radiation doses (3 mSv and 0.5 mSv) with various tube voltages (70 kVp/100 kVp/120 kVp) and algorithms (DLIR, DLIR-MAR, ASIR-V, and ASIR-V MAR) (Median [Q1,Q3])

| **Voltage, algorithm** | **0.5mSv** | **3mSv** | **P** |
| --- | --- | --- | --- |
| **SNR** |  |  |  |
| **70kVp ASIR-V** | **7.4 [6.1, 9.6]** | **10.6 [7.7, 18.4]** | 0.059 |
| **70kVp ASIR-V MAR** | **8.9 [8.5, 10.2]** | **18.5 [17.5, 20.5]** | **＜0.001** |
| **70kVp DLIR-H** | **6.47 [5.69, 9.30]** | **7.5 [6.5, 18.6]** | 0.211 |
| **70kVp DLIR-H MAR** | **9.1 [7.3, 13.6]** | **17.8 [14.5, 25.2]** | **＜0.001** |
| **100kVp ASIR-V** | **7.7 [6.7, 8.7]** | **12.7 [9.8, 16.9]** | **0.006** |
| **100kVp ASIR-V MAR** | **9.5 [8.2, 12.0]** | **15.4 [12.8, 23.0]** | **0.001** |
| **100kVp DLIR-H** | **7.0 [5.9, 9.4]** | **10.8 [9.6, 17.7]** | **0.019** |
| **100kVp DLIR-H MAR** | **9.4 [6.9, 13.5]** | **17.1 [11.9, 25.8]** | **0.006** |
| **120kVp ASIR-V** | **8.8 [8.1, 9.21]** | **14.9 [11.7, 20.6]** | **＜0.001** |
| **120kVp ASIR-V MAR** | **10.5 [8.4, 11.8]** | **18.7 [16.0, 21.8]** | **＜0.001** |
| **120kVp DLIR-H** | **7.9 [6.7, 9.8]** | **16.5 [11.1, 20.7]** | **0.001** |
| **120kVp DLIR-H MAR** | **10.3 [8.0, 13.2]** | **20.7 [14.2, 26.6]** | **0.002** |

SNR, signal-to-noise ratio; ASIR-V, 50% adaptive statistical iterative reconstruction-V; ASIR-V MAR, ASIR-V 50% with MAR; DLIR-H, deep learning image reconstruction with high strength; DLIR-H MAR, DLIR-H with MAR;
