# Supplementary material for: Metal artifact reduction combined with deep learning image reconstruction algorithm for CT image quality optimization: a phantom study
Source: PeerJ. 2025 Jun 4;13:e19516. doi: 10.7717/peerj.19516 (PMC12145087; doi:10.7717/peerj.19516)
Supplement: Supplemental Information 6 [file peerj-13-19516-s006.docx]

**Supplemental Table 2** Statistical analysis of AI under different radiation doses (3 mSv and 0.5 mSv) with various tube voltages (70 kVp/100 kVp/120 kVp) and algorithms (DLIR, DLIR-MAR, ASIR-V, and ASIR-V MAR) (Median [Q1,Q3])

| **Voltage, algorithm** | **0.5mSv** | **3mSv** | **P** |
| --- | --- | --- | --- |
| **AI** |  |  |  |
| **70kVp ASIR-V** | **113.0 [98.8, 124.0]** | **103.5 [85.1, 219.3]** | 0.882 |
| **70kVp ASIR-V MAR** | **46.1 [33.5, 54.9]** | **39.4 [35.0, 48.3]** | 0.734 |
| **70kVp DLIR-H** | **115.6[104.3,124.3]** | **92.4 [81.4,111.0]** | **＜0.001** |
| **70kVp DLIR-H MAR** | **43.1 [36.6, 48.3]** | **31.9 [30.2, 38.4]** | **＜0.001** |
| **100kVp ASIR-V** | **67.8 [61.7, 77.7]** | **115.0 [68.6, 221.5]** | **＜0.001** |
| **100kVp ASIR-V MAR** | **38.3 [20.7, 50.9]** | **29.1 [24.6, 35.6]** | 0.093 |
| **100kVp DLIR-H** | **79.6 [68.8, 83.5]** | **72.6 [59.4, 94.3]** | 0.734 |
| **100kVp DLIR-H MAR** | **39.7 [29.8, 46.5]** | **27.9 [25.0, 33.2]** | **＜0.001** |
| **120kVp ASIR-V** | **65.3 [56.2, 78.6]** | **75.7 [58.7, 99.4]** | 0.062 |
| **120kVp ASIR-V MAR** | **38.2 [4.5, 44.5]** | **29.2 [25.8, 34.9]** | **＜0.001** |
| **120kVp DLIR-H** | **71.2 [66.5, 80.7]** | **60.7 [50.3, 66.5]** | **＜0.001** |
| **120kVp DLIR-H MAR** | **37.3 [28.7, 46.0]** | **27.3 [23.3, 31.4]** | **0.001** |

AI, artifact index; ASIR-V, 50% adaptive statistical iterative reconstruction-V; ASIR-V MAR, ASIR-V 50% with MAR; DLIR-H, deep learning image reconstruction with high strength; DLIR-H MAR, DLIR-H with MAR;
