# Supplementary material for: Metal artifact reduction combined with deep learning image reconstruction algorithm for CT image quality optimization: a phantom study
Source: PeerJ. 2025 Jun 4;13:e19516. doi: 10.7717/peerj.19516 (PMC12145087; doi:10.7717/peerj.19516)
Supplement: Supplemental Information 7 [file peerj-13-19516-s007.docx]

**Supplemental Table 3** Statistical analysis of Noise under different radiation doses (3 mSv and 0.5 mSv) with various tube voltages (70 kVp/100 kVp/120 kVp) and algorithms (DLIR, DLIR-MAR, ASIR-V, and ASIR-V MAR) (Median [Q1,Q3])

| **Voltage, algorithm** | **0.5mSv** | **3mSv** | **P** |
| --- | --- | --- | --- |
| **Noise** |  |  |  |
| **70kVp ASIR-V** | **67.4 [62.6, 68.9]** | **32.9 [32.1, 35.1]** | **＜0.001** |
| **70kVp ASIR-V MAR** | **63.8 [62.6, 66.0]** | **35.5 [33.0, 36.8]** | **＜0.001** |
| **70kVp DLIR-H** | **37.3 [36.5, 38.3]** | **17.8 [17.2, 18.9]** | **＜0.001** |
| **70kVp DLIR-H MAR** | **38.8 [37.0, 40.2]** | **18.9 [18.1, 20.2]** | **＜0.001** |
| **100kVp ASIR-V** | **59.9 [58.0, 63.4]** | **28.4 [28.1, 29.1]** | **＜0.001** |
| **100kVp ASIR-V MAR** | **58.4 [56.8, 60.4]** | **32.5 [30.4, 33.4]** | **＜0.001** |
| **100kVp DLIR-H** | **36.4 [35.1, 40.8]** | **15.7 [15.2, 16.0]** | **＜0.001** |
| **100kVp DLIR-H MAR** | **37.3 [36.3, 39.5]** | **17.0 [16.2, 17.9]** | **＜0.001** |
| **120kVp ASIR-V** | **60.9 [58.4, 63.1]** | **29.9 [29.6, 31.0]** | **＜0.001** |
| **120kVp ASIR-V MAR** | **60.6 [58.6, 62.6]** | **30.5 [29.6, 32.2]** | **＜0.001** |
| **120kVp DLIR-H** | **38.2 [34.6, 40.8]** | **17.2 [16.1, 17.7]** | **＜0.001** |
| **120kVp DLIR-H MAR** | **38.2 [36.0, 40.3]** | **16.6 [16.1, 17.4]** | **＜0.001** |

ASIR-V, 50% adaptive statistical iterative reconstruction-V; ASIR-V MAR, ASIR-V 50% with MAR; DLIR-H, deep learning image reconstruction with high strength; DLIR-H MAR, DLIR-H with MAR
